# Repeated large-scale forest inventory data from an unmanaged European beech forest at Hainich National Park, Germany

**DOI:** 10.1038/s41597-026-08215-x

**Published:** 2026-08-29

**Authors:** Markus Erhard Schorn, Jürgen Bauhus, Christian Wirth, Nadja Rüger

**Affiliations:** 1 German Centre for Integrative Biodiversity Research (iDiv) Halle-Jena-Leipzig, Leipzig, Germany; 2 Leipzig University, Faculty of Economics, Leipzig, Germany; 3 University of Freiburg, Chair of Silviculture, Freiburg, Germany; 4 Leipzig University, Faculty of Life Sciences, Leipzig, Germany; 5 Max-Planck-Institute for Biogeochemistry, Jena, Germany

## Abstract

This dataset contains forest inventory data from four censuses (1999, 2007, 2013 and 2023) from a single ~28 ha permanent monitoring plot in a beech-dominated forest that has been unmanaged since 1965 at Hainich National Park, Thuringia, Germany. For each census, the location, species identity and diameter at breast height (dbh) of all living trees with a dbh ≥1 cm was recorded. Additional attributes for living trees include vitality, crown coverage by neighboring trees, sociological class, and observed damages. For trees that died since the previous census, information on cause of mortality, degree of decay and stem integrity is provided for some censuses. This dataset enables analyses of the dynamics of unmanaged Central European beech-dominated forests over more than two decades.

## Background & Summary

Forests dominated by European beech (*Fagus sylvatica* L.) are among the most common forest types in Central Europe and represent the potential natural vegetation across large areas of the region^1^. True primeval beech forests are absent in Germany and scarce across Central Europe^2^. Nevertheless, forests that have been unmanaged or managed at low intensity in recent decades can serve as a suitable substitute to study the near-natural dynamics of these forests, the impacts of close-to-nature forestry^3^, or their conservation value^4^. In this light, a large-scale and long-term forest monitoring plot was established at Weberstedter Holz in the core zone of Hainich National Park in 1999. Here, we present forest inventory data from four repeated inventories (1999, 2007, 2013 and 2023) at the ~28 ha plot.

The forest in this area can be described as a *Hordelymo-Fagetum*^5,6^. European beech is the dominant tree species, with common ash (*Fraxinus excelsior* L.), European hornbeam (*Carpinus betulus* L.), Sycamore maple (*Acer pseudoplatanus* L.) and Norway maple (*Acer platanoides* L.) admixed. Single trees of elm (*Ulmus glabra* Huds.), pedunculate oak (*Quercus robur* L.), field maple (*Acer campestre* L.), wild cherry (*Prunus avium* L.), lime (*Tilia spec*.), and aspen (*Populus tremula* L.) can also be found.

Until the end of the 19^th^ century, the forest in the area was primarily managed by local communities as a coppice-with-standards, with beech, ash, maple, oak, hornbeam and elm as the main tree species. During the first half of the 20^th^ century, the stand underwent a transition to a high forest with selective harvesting, resulting in relatively low timber extraction rates (2–3.7 m^3^/ha/year). Forest management activities largely ceased in 1965, when the area was designated as a military training ground. In 1997, the area became a National Park. For a comprehensive account of the area’s management history, refer to two disserations^5,7^.

The dataset contains information on the dbh, spatial coordinates, species affiliation, and sociological status for all living stems across all inventory years. In 2007, 2013, and 2023, information on the vitality, coverage by other tree crowns, and visible damages is available for living trees. For trees that died since the previous inventory, information on the cause of mortality, structural integrity (complete or fragmented), and position (lying or standing) of the dead stem is available in 2007, 2013, and 2023. In 2007 and 2013, information on the decay stage of dead stems is available.

In the past, the data have been used to study gap and regeneration dynamics^5^, carbon pools^7^, mortality causes^8^, effects of neighborhood diversity on forest productivity^9^, drivers of tree height heterogeneity^10^, or to parametrize a demographic forest model^11^. The data can also be used to study forest dynamics over time, drought effects on demographic rates and carbon dynamics, including their interactions with neighborhood characteristics, and be included in comparative analyses, e.g., through the European Forest Reserves Initiative EuFoRIa (https://www.wsl.ch/de/projekte/euforia), where the plot is registered.

## Methods

### Study site

The plot is located 15 kilometers north-east of Eisenach in the Hainich National Park (Thuringia), Germany. The centroid of the plot is at N 51° 4′ 47.74, E 10° 26′ 45.10 (WGS84). The plot is situated at 425–455 m above sea level and north-east exposed with a gentle slope of less than 5° for the most part^10^. Soils at the study area are generally Luvisols developed from Pleistocene loess over Triassic limestone^7^. The plot originally covered 28.5 ha. For the 2023 inventory, the southern boundary was fixed at gy = 30 (northing_m = 5661368.533) to exclude an abandoned road, resulting in a plot of 27.624 ha in size. Consequently, the main dataset in this publication contains data corresponding only to the 27.624-ha area across all inventory years. However, we also provide additional data from the truncated southern edge, such that the data used in previous publications can be recreated^8,9^.

**Fig. 1:**
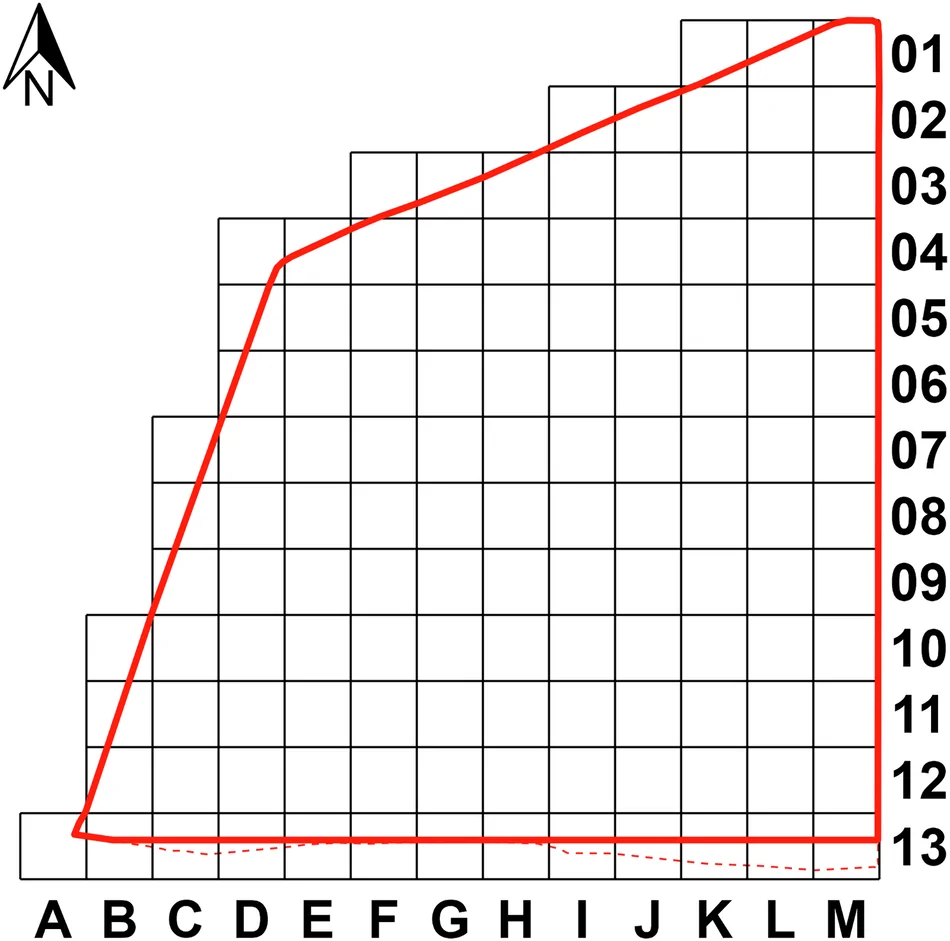
Plot and subplot boundaries along with the naming convention for subplots. The solid red line represents plot boundaries applicable for this dataset, the solid black lines represent the 50 × 50 m subplots and the dashed red line represents the former southern plot boundary.

The plot consists of 50 × 50 m subplots that are aligned with the main cardinal directions. Rows are north–south oriented and numbered consecutively from 01 to 13, while columns are west–east oriented and labeled alphabetically from A to M. Consequently, subplot A13 represents the southwestern corner of the plot, and subplot M01 the northeastern corner. As the overall plot is irregular in shape due to topographic constraints, not all subplots are fully contained within the plot boundaries (Fig. 1).

In 2000/2001, 20 fenced areas of about 140 m² each were established as wildlife exclosures inside the plot to study natural tree regeneration under reduced browsing. The cumulative fenced area is 2,864 m². Detailed information on single fenced areas can be found in the metadata of this dataset. The fences were demolished between 2007 and 2013, such that all fences were found dysfunctional in the 2013 inventory. The fences were completely removed after 2013.

### Tree measurements

All woody stems ≥1 cm dbh were marked and spatially located by recording their distance and azimuth to the edges of the subplots or other previously located trees as reference points. For all stems, the status was recorded (alive, dead, missing) and the dbh was measured with millimeter precision using measurement tapes. In each inventory, the data was collected by different field crews with little prior knowledge of the plot. Stems were usually measured at 1.3 m height (breast height). In 2023, deviations of the measurement height were recorded. In 1999, 2007, and 2013 information about deviations is not available. In all inventories, all stems above the respective dbh threshold were measured. Multiple stems ≥1 cm dbh that belong to the same individual share the same coordinates and can thereby be identified. In the 1999 and 2007 inventories, there may be some unidentified multiple stems recorded as separate individuals. Additionally, multiple stems ≥1 cm dbh of the same individual were, apparently, not always measured then.

In 1999, 25 trees were not recorded or recorded as dead, but were found to be alive in 2007 (possibly because the fieldwork in 1999 was conducted outside of the growing season when live/dead classification of trees is less reliable). For these trees, their dbh in 1999 was estimated based on light availability at the location. All remarks were checked to flag these trees (“dbh_1999_estimated”), but it cannot be assured that all of them were captured. In the 2007 and 2013 inventories, field crews had information on previously recorded dbh of the trees. Thus, in case of apparent negative growth, they re-measured the tree, introducing a positive bias on growth estimates. After the 2007 and 2013 inventories, 159 trees were re-visited to check discrepancies between their recorded dbh in 1999 and 2007 or their status (dead, alive). Often, these were due to wrong decimal places or transcription errors and were corrected. The original values were not preserved. These cases are flagged (“dbh_2007_estimated”, “dbh_2013_estimated”).

All trees inside fences or formerly/later fenced areas are flagged (“inside_fence”). However, coordinates of fences and trees were associated with some uncertainty and the classification as “inside_fence” may not be fully correct. As fences were only operational between 2000 and ~2010, this information can be neglected for the 1999 and largely also for the 2023 inventory. It is mainly important when estimating the number of recruits because most recruits of the 2013 inventory are inside fenced areas. In the first two censuses, exact dates of observations were not recorded, and only the entire inventory period can be assigned (September-December 1999, May/June 2007). The parameters vitality, coverage, damage, cause of death, and type of dead tree (integrity and position of dead stems) were only recorded in 2007, 2013, and 2023. See Holzwarth *et al*.^8^ for more information on the cause of death and Mueller-Using & Bartsch^12^ for more information on the “dead_type”. The sociological status (“kraft”) was recorded in all inventories and refers to the social class according to Kraft^13^. The decay stage was only recorded in 2007 and 2013. See Mueller-Using & Bartsch^12^ for more information.

## Data Records

The main dataset is stored as three separate csv files on the iDiv biodiversity data portal and is available under https://doi.org/10.25829/idiv.3597-5sy386 or at https://idata.idiv.de/ddm/Data/ShowData/3597^14^. The file *hainich_inventory.csv* contains the inventory data, the file *hainich_metadata_01_column_definition.csv* contains information on the names, definitions, data types, and units of all columns, and the file *hainich_metadata_02_categories*.csv contains information on all possible categories and codes in all parameters.

The additional file *hainich_southern_edge.csv* contains data from the first three censuses from the truncated southern part of the plot. We recommend not to use this data for future research but only to replicate the data used in previous publications. The additional file *hainich_remeasured_subplots_2023.csv* contains second observations from subplots that were measured twice in the 2023 census (see below). We recommend to use this data only to determine measurement errors.

All files are UTF-8 encoded. Similar to the ForestGEO format^15^, each stem is represented by one row in each inventory with the status “P” (prior) in inventories before the first inclusion of the stem, status “A” (alive) if the stem was found alive in the respective inventory, status “D” (dead) in all inventories after the death of the stem and status “M” (missing) if the stem was found alive in a previous census but not relocated in the current census, indicating that the fate of the stem could not be confirmed.

## Data Overview

**Fig. 2:**
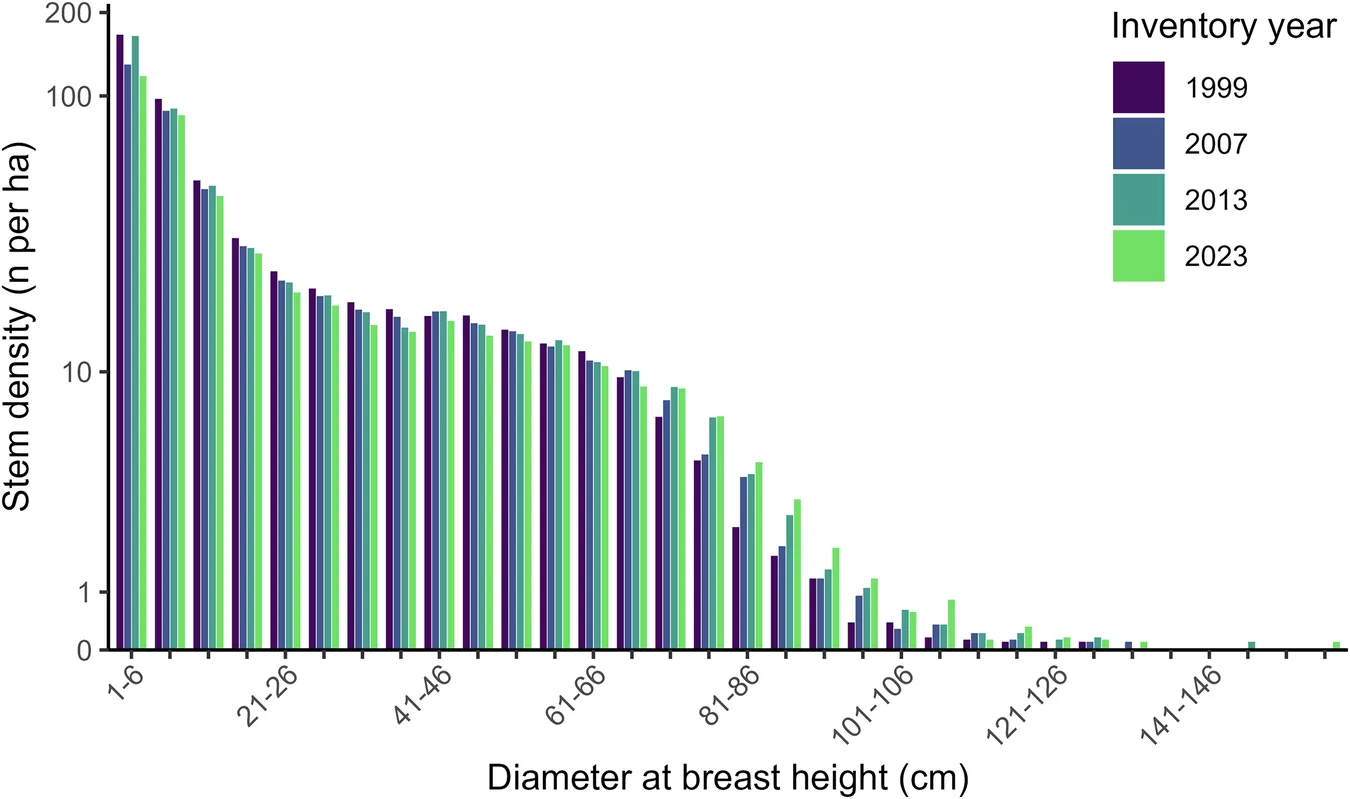
Diameter distribution of all living stems in the four inventories. Diameters are binned in 5 cm-classes.

Overall, the dataset contains information from 17438 individual stems from 14 woody species that were alive and larger than the dbh threshold of 1 cm in at least one of the four inventories. This corresponds to 519 living stems/ha in 1999, 465 living stems/ha in 2007, 505 living stems/ha in 2013, and 439 living stems/ha in 2023. The basal area is 36.2 m²/ha in 1999, 37.5 m²/ha in 2007, 39.9 m²/ha in 2013, and 39.5 m²/ha in 2023. The size distribution follows an inverse-J shape with a hump for trees between ~40 and 80 cm dbh. The number of large trees >70 cm dbh increases over time while the number of trees <70 cm generally decreases (Fig. 2). The share of beech is about 90% in terms of stem numbers and about 70% in terms of basal area.

## Technical Validation

In 2023, a total of 1089 stems in an area of 2 ha (eight 50 × 50 m subplots) were inventoried twice within 1–7 weeks (median: 2 weeks) by different teams to determine the error in the tree status assignment and in the dbh measurement. 931 trees (85.5%) were found alive or dead in both observations (Table 1). Only 13 of these were classified as alive in one observation and dead in the other. Thus, tree status assignment seems to be reliable. However, 98 trees were not found (status “M”) in the first observation and 144 in the second observation. Of these, 88 (first observation, 89.8%) or 101 (second observation, 70.1%) were <5 cm dbh in the 2013 inventory. Only 5 (first observation, 5.1%) or 19 (second observation, 13.2%) of all trees with status “M” were found to be alive in the respectively other observation. Thus, around 90.9% of the missing trees could be considered to be dead. In the main dataset, the first observation was used for all re-measured trees. The second observation is stored in the file *hainich_remeasured_subplots_2023.csv*.

**Table 1:** Stem statuses from the two observations of stems that were re-measured during the 2023 inventory.

			Second observation		
			Alive	Dead	Missing
		**total**	**811**	**134**	**144**
**First observation**	**Alive**	**830**	802	9	19
	**Dead**	**161**	4	116	41
	**Missing**	**98**	5	9	84

**Fig. 3:**
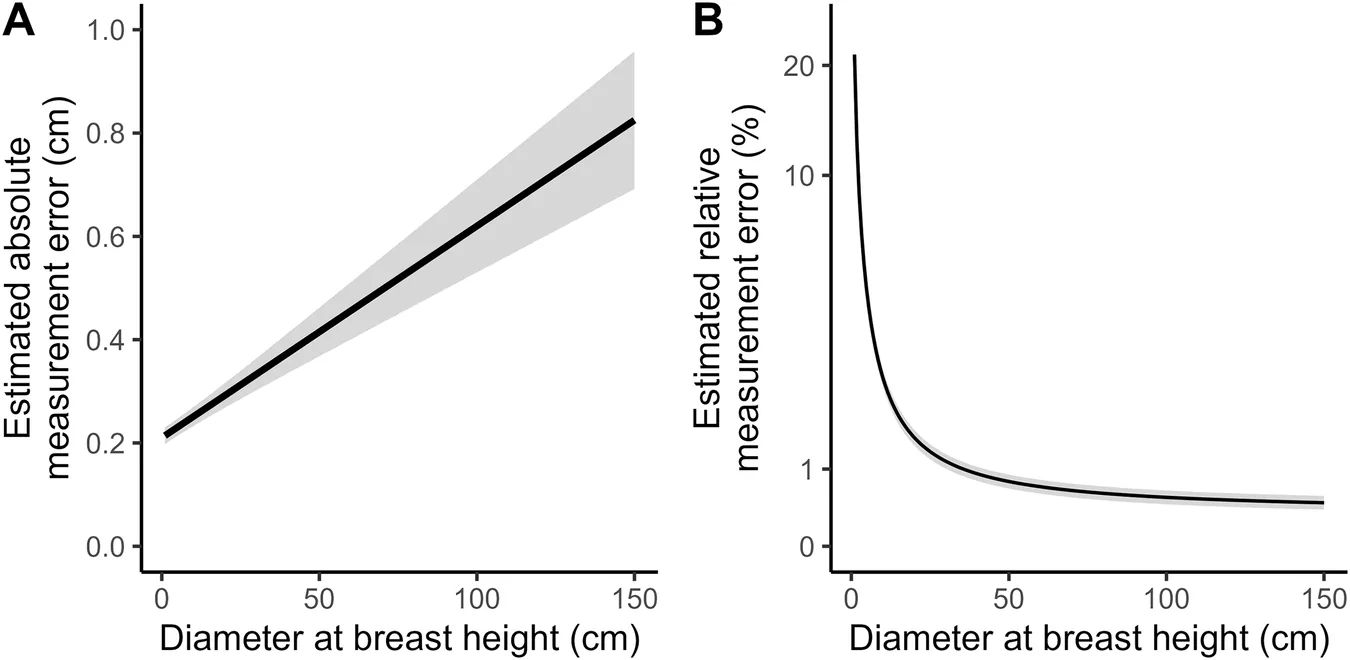
Estimated absolute (**A**) and relative (**B**) small error (SD_1_) in the measurement of the diameter at breast height (dbh) as a function of the latter. The black line represents the estimated error. The grey area represents the 95% confidence interval.

We determined the measurement error in the dbh measurement following the approach of Chave *et al*.^16^ and Rüger *et al*.^17^. That means, we used a Bayesian model to fit two normal distributions to describe the difference between the measured dbh and the estimated true dbh of each tree. The first normal distribution describes small errors and has a standard deviation proportional to the diameter (SD_1_ = sda + sdb × dbh) while the second one describes larger errors such as, e.g., typos or decimal errors and has a constant standard deviation (SD_2_). The 795 double-blind re-measurements of living trees were best fit with SD_1_ = 0.2139 + 0.0041 × dbh (Fig. 3) and SD_2_ = 8.34 cm, with 2% of the trees subject to the larger error. For example, the measured dbh of a 50 cm tree has a typical error of 0.42 cm (98% probability) or of 8.34 cm (2% probability).

## Data Overview

The dataset is available in the iDiv Data Portal under https://doi.org/10.25829/idiv.3597-5sy386 or at https://idata.idiv.de/ddm/Data/ShowData/3597^14^.

## Data Availability

The code used to clean and consolidate the published dataset from the raw data is available at https://git.idiv.de/ms13xava/hainich_data_public.
